# Inflammation Modulating Activity of the Hydroethanol Stem Bark Extract of *Bombax costatum* in Murine Models

**DOI:** 10.1155/2022/6882147

**Published:** 2022-08-22

**Authors:** Meshack Antwi-Adjei, Kofi Oduro Yeboah, James Oppong-Kyekyeku, Newman Osafo

**Affiliations:** ^1^Department of Pharmacology, University of Cape Coast, Cape Coast, Ghana; ^2^Department of Pharmacology, Kwame Nkrumah University of Science and Technology, Kumasi, Ghana; ^3^Department of Pharmaceutical Chemistry, Kwame Nkrumah University of Science and Technology, Kumasi, Ghana

## Abstract

*Bombax costatum* (Bombacaceae) is traditionally used as a decoction of the leaves, stem, and root to treat headaches, fever, and oedema that may be associated with inflammatory conditions. Thus, the aim of this study was to evaluate the effect of 70%^v^/_v_ ethanolic extract of the stem bark of *Bombax costatum* on acute and chronic inflammation. The effect of *Bombax costatum* extract (10, 50, 100 mg kg^−1^, p.o) was studied in prostaglandin E_2_-induced paw oedema in Sprague–Dawley rats (*n* = 5). Subsequently, the effect of the extract on clonidine and haloperidol-induced catalepsy was also investigated in ICR mice (*n* = 5). Finally, the ability of the extract to inhibit chronic inflammation was studied using a rat adjuvant-induced arthritis model. Pre-emptive and therapeutic administration of the extract at all doses significantly suppressed the formation of oedema following prostaglandin *E*_2_ administration. As a measure of indirect antihistaminic effect, treatment with the extract suppressed clonidine-induced catalepsy but not haloperidol-induced catalepsy. Moreover, *Bombax costatum* extract significantly inhibited joint inflammation and damage following injection of complete Freund's adjuvant. Treatment with the extract also inhibited the onset of polyarthritis; thus, suppressing the systemic spread of joint inflammation from ipsilateral limbs to contralateral limbs. In conclusion, the hydroethanol extract of the stem bark of *Bombax costatum* inhibits both acute and chronic inflammation.

## 1. Introduction

Inflammation, in its primordial sense, is a beneficial immune response targeted at eliminating offending or phlogistic agents such as bacteria, viruses, and tissue injury [1, 2]. The acute phase of this response is characterized by the rapid influx of granulocytes, immediately followed by the maturation of monocytes into inflammatory macrophages with subsequent functional alteration of resident tissue macrophages [1]. The process is self-limiting and abates once the initiating noxious stimuli are removed via phagocytosis [3]. Nonetheless, the inflammatory response is sometimes dysregulated leading to chronic, persistent inflammation that results in scarring and loss of organ function [4]. The dysregulation and persistence of the inflammatory process play a critical role in the development and progression of many chronic diseases including cancer and autoimmune diseases such as rheumatoid arthritis [1].

Currently, conventional drugs used in the treatment or management of inflammatory disorders include nonsteroidal anti-inflammatory drugs (NSAIDs), disease-modifying antirheumatoid drugs (DMARDs), and glucocorticoids [5]. Despite the benefit of providing symptomatic relief and slowing the progression of chronic inflammatory disorders (mostly DMARDs), these agents do not ultimately stop the progression of inflammatory diseases, such as arthritis. Moreover, doses associated with maximal efficacies of DMARDs are also associated with significant toxicities; thus, limiting the clinical benefit of these agents [6]. Likewise, NSAIDs and glucocorticoids used in the management of acute inflammatory disorders and flares in chronic inflammation are associated with significant adverse effects such as gastrointestinal bleeding and immunosuppression, respectively [7]. This highlights the need for newer agents that are more efficacious and less toxic, including those from natural sources.

*Bombax costatum* (Bombacaceae), locally called the red-flowered silk-cotton tree, may be one of such natural sources. The plant is largely distributed in the savanna, dry woodlands from Senegal through West Africa to Southern Chad. This deciduous tree is covered with thick, corky bark and reaches up to about 30 m in height and 100 cm in diameter. The leaves are digitally compound, ovate with 5–7 leaflets and petioles 8–15 cm long. The flowers are solitary, bisexual and 5–7 cm with deep red, orange, or yellow, tulip-shaped, glabrous peduncles. The fruits, contained in ellipsoidal dark-brown capsules, are embedded in white floss called kapok and contain several small seeds [8]. Traditionally, the root decoction is used in the management of epilepsy whereas decoction of the leaves is used in the treatment of fever, headaches, leucorrhoea, diarrhoea, convulsion, and jaundice [9]. Furthermore, stem bark decoction is used in the treatment of skin diseases, trichomoniasis, amoebiasis, and oedema.

As part of our continuous research into medicinal plants, this study aims at establishing the possible anti-inflammatory activity of the hydroethanol extract of the stem bark of *Bombax costatum* in adjuvant-induced arthritis. In addition, the effect of the extract on specific mediators of inflammation is investigated in prostaglandin E_2_-induced oedema and catalepsy models.

## 2. Materials and Methods

### 2.1. Plant Collection and Extraction

The stem bark of *Bombax costatum* was collected from Nkawkaw in the Eastern Region of Ghana between February and April 2019. The bark was subsequently authenticated at the Department of Herbal Medicine, Kwame Nkrumah University of Science and Technology, and a voucher specimen KNUST/HM1/2019/WP006 was deposited in the herbarium of the Department of Herbal Medicine, KNUST. To prepare a hydroethanol extract, the stem bark of the plant was air-dried for 14 days after which it was milled into a coarse powder using a hammer mill (DF-15, DADE, 15 kg/h-110v, HXJQ, China). Afterwards, 600 g of the powder was extracted by cold maceration in 2.0 L of 70% (^v^/_v_) ethanol for 72 h following which the supernatant was decanted and filtered. The filtrate was then concentrated at 60°C under low pressure in a rotary evaporator (RE-LA-5, LAB-GENI, China), to obtain a dark-brown liquid which was evaporated to dryness in an oven (MOV-112PE, HXJQ, Panasonic, China) at 60°C over 24 h. The semisolid concentrate was stored in a desiccator to remove excess moisture. A final percentage yield of 8.76% (^w^/_w_) was obtained. Prior to administration throughout the study, the extract was freshly reconstituted in 2% ^w^/_v_ tragacanth mucilage (dissolved in normal saline), herein referred to as hydroethanol*Bombax costatum* extract (BCE).

(1) $$\begin{array}{c} \%\text{Yield}=\frac{M_{1}}{M_{0}}x100\%, \end{array}$$

where *M*_*0*_ was the mass of the stem bark sample and *M*_*1*_ was the mass of the crude extract.

### 2.2. Animals

Sprague–Dawley rats (200–250 g) and ICR mice (25–30 g) of both sexes were purchased from Noguchi Memorial Institute for Medical Research, University of Ghana, Accra, Ghana. All animals were housed in the Animal facility of the Department of Pharmacology, KNUST. In accordance with Animal Welfare Regulations and the Public Health Service Policy on Humane Care and Use of Laboratory Animals (PHS 2002), all animals used this study were humanely handled throughout the experimental period. Moreover, studies on the rodents were conducted with the approval of the Department of Pharmacology, KNUST Ethics Committee. The animals were randomly grouped (*n* = 5) and housed in stainless steel cages (34 cm × 47 cm × 18 cm) with softwood shavings as bedding and were fed with a normal commercial pellet diet (GAFCO, Tema, Ghana). All animals were given access to water. The animals were allowed enough time to acclimatize to the new environment and were maintained at a room temperature of 26 ± 2°C in a 12 h light-dark cycle. Each animal was used only once and at the end of each experiment, all animals were euthanized.

### 2.3. Chemicals and Reagents

Dexamethasone (Pharm-Inter, Brussels, Belgium), methotrexate (Pharm-Inter, Brussels, Belgium), diclofenac (Trogue, Hamburg, Germany), complete Freund's adjuvant (CFA) (Sigma-Aldrich, St. Louis, MO, USA), clonidine (Teva Ltd, Wakefield, UK); Haloperidol (Alkem, Mumbai, India), chlorpheniramine maleate (Unimark Remedies Ltd, Mumbai, India), and prostaglandin *E*_2_ (Boster, CA, USA).

### 2.4. Microorganism

Heat-killed *Mycobacterium tuberculosis* (strains C, DT, and PN (mixed)) was a kind donation from the Ministry of Agriculture, Fisheries and Food, UK.

### 2.5. Prostaglandin E_2_-Induced Paw Oedema in Rats

Paw oedema was induced by subplantar injection of 0.2 ml (1 nM) prostaglandin *E*_2_ into the right hind limb of the rats (200–250 g, *n* = 5) [10]. Rats were treated with either normal saline (1 ml kg^−1^, p.o.), diclofenac (30 mg kg^−1^ p.o.), or BCE (10, 50, 100 mg kg^−1^ p.o.) therapeutically. Using water displacement plethysmography, paw volume was measured before and after PGE_2_ induction, at 30 min intervals over 3 h. Oedema was estimated from the percentage change in paw volume over the different time points. The increase in paw volume was expressed using the formula:

(2) $$\begin{array}{c} \%\text{Change in paw volume}=\left[\frac{\left(PW_{0}-PW_{t}\right)}{PW_{0}}\right]x100, \end{array}$$

where, *PW*_*0*_ and *PW*_*t*_ represent paw volume before and at time, *t* post PGE_2_ injection. Total oedema cumulatively induced over 3 h was determined as the area under the time-course curve (AUC), and the percentage inhibition of the total oedema for each treatment was calculated using the formula as follows:

(3) $$\begin{array}{c} \%\text{Inhibition of oedema}=\left[\frac{\text{AUC control}-\text{AUC treated}}{\text{AUC control}}\right]\times 100. \end{array}$$

### 2.6. Clonidine-Induced Catalepsy in Mice

Employing the bar test, the indirect antihistaminic activity of BCE was demonstrated in clonidine-induced catalepsy, defined as maintenance of an imposed posture for a long time before regaining normal posture [11]. Briefly, clonidine (1 mg kg^−1^) was injected subcutaneously into ICR mice (25–30 g, *n* = 5) and their forepaws were placed on a horizontal bar (1 cm in diameter, 3 cm above a table). The time taken for each animal to remove their paws from the bar, a measure of catalepsy, was recorded and the duration of catalepsy was measured at 30-minute intervals for a total duration of 3 h. Mice received either BCE (10–100 mg kg^−1^, p.o, 1 h), chlorpheniramine maleate (5 mg kg^−1^, ip, 30 min), or 1 ml kg^−1^, p.o of normal saline after catalepsy induction.

### 2.7. Haloperidol-Induced Catalepsy in Mice

To induce catalepsy, haloperidol (1 mg kg^−1^) was injected subcutaneously into ICR mice (25–30 g, *n* = 5) and their forepaws were placed on a horizontal bar as described in section 2.6. The time taken for each animal to remove their paws from the bar, a measure of catalepsy, was recorded and the duration of catalepsy was measured at 30-minute intervals for a total duration of 3 h. Mice received either BCE (10–100 mgkg^−1^, p.o, 1 h) or normal saline (1 ml kg^−1^, p.o.) after catalepsy induction.

### 2.8. Chronic Inflammation

#### 2.8.1. Induction of Rat Adjuvant-Induced Arthritis

Adjuvant arthritis was induced as previously described by Pearson [12]. Briefly, 100 *µ*l of complete Freund's adjuvant (CFA), prepared as a suspension of 5 mg ml^−1^ of heat-killed *Mycobacterium tuberculosis* (strains C, DT, and PN (mixed)) in paraffin oil, was injected subplantar into the right hind paw of the rats. Nonarthritic control group received only intraplantar injection of 100 *µ*l of sterile paraffin oil referred to as incomplete Freund's adjuvant (IFA). Foot volume was measured with a plethysmometer (Ugo Basile S.R.L., Varese, Italy) for both the injected (ipsilateral) and noninjected (contralateral) hind paws prior to intraplantar injection of CFA/IFA (day 0) and daily for 28 days [13]. The oedema component of inflammation was quantified by measuring the difference in foot volume between day 0 and the various time points.

Paw volumes were individually normalized as a percentage of change from their values at day 0 and then averaged for each treatment group. Total oedema induced was measured as the area under the time-course curve (AUC). In a curative study, rats received either vehicle (normal saline), extract (10, 50, and 100 mg kg^−1^, p.o, daily), dexamethasone (3.0 mg kg^−1^, ip every other day), or methotrexate (1.0 mg/kg, ip every 4 days) after induction of oedema on day 14 until the 28^th^ day. All drugs were freshly prepared on each day of drug administration.

#### 2.8.2. Arthritic Indices and Radiological Assessment

A radiological assessment of the severity of cartilage and bone destruction was done on the 29^th^ day. Briefly, rats were anaesthetized with pentobarbitone sodium (20 mg kg^−1^, ip) after which radiographs of the hind paw were taken with an X-ray apparatus [Softex, Tokyo, Japan] and industrial *X*-ray film (Fuji Photo Film, Tokyo, Japan), at a peak voltage of 30-kV with a 10-s exposure, and a tube-to-film distance of 45 cm for lateral projections. The severity of bone and cartilage damage was blindly scored based on the degree of bone destruction, paw oedema, osteoporosis, and the extent of osteophyte formation [14]. Scoring was done on a scale of 0–3 (0–no degenerative joint changes; 1, slight soft tissue oedema, joint space narrowing, osteolysis, subchondrial erosion, degenerative joint changes; 2, mild to moderate soft tissue oedema, joint space narrowing, osteolysis, subchondrial erosion, degenerative joint changes; 3, severe soft tissue oedema, joint space narrowing, osteolysis, subchondrial erosion, degenerative joint changes).

### 2.9. Statistical Analysis

All data are presented as Mean ± SEM. The time-course curves for changes in paw volume were subjected to a two-way (treatment *x* time) repeated measures analysis of variance with Tukey's post hoc test. Differences in AUCs were analysed by one-way ANOVA followed by Tukey's post hoc test. All graphs were plotted using GraphPad Prism for Windows Version 8.0.1 (GraphPad, San Diego, CA).

## 3. Results

### 3.1. Effect of Extract on PGE_2_-Induced Paw Oedema

**Figure 1:**
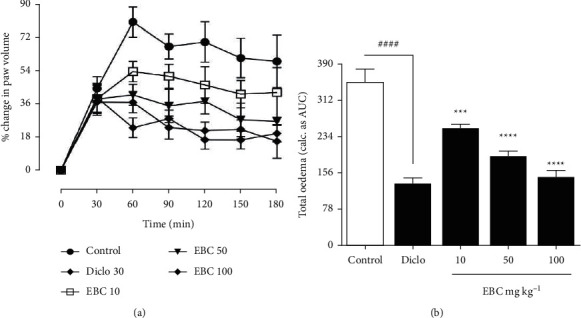
*Bombax costatum* extract inhibits prostaglandin E_2_-induced oedema in rats. Sprague–Dawley rats (200–250 g) were injected with 200 *µ*L 1 *n*M PGE_2_ into the subplantar tissue of the right hind paw (a) Total oedema induced during the 3 h period was calculated as area under the time-course curves, AUC (b) Data are presented as Mean ± S.E.M. (*n* = 5). ^####^*p* < 0.0001, diclofenac-treated compared to vehicle-treated; ^*∗∗∗*^*p* < 0.001 and ^*∗∗∗∗*^*p* < 0.0001 extract-treated compared to vehicle-treated.

Increased release of prostaglandin *E*_2_ during acute inflammation contributes to capillary permeability and oedema formation. Doses of the extract were chosen based on the results of an acute toxicity study by Ambi et al. [15]. In this study, the median lethal dose via oral route was greater than 5000 mg/kg in albino rats. The authors then decided to select doses less than one-fourth of the LD_50_ to further reduce any risk of toxicity. From the study, treatment with diclofenac (10, 50, 100 mg kg^−1^), following the PGE_2_ challenge, significantly (*p* < 0.0001) reduced mean oedema formation over 3 h to 20.43 ± 4.55% compared to 54.48 ± 9.98% of the saline-treated control rats (Figures 1(a) and 1(b)). Compared to the saline-control, the extract at doses of 10, 50, and 100 mg kg^−1^ similarly reduced paw swelling significantly *p* < 0.0001, with mean paw volumes of 39.02 ± 6.80%, 29.37 ± 5.32%, and 22.30 ± 4.80%, respectively (Figure 1(a)). Both the extract and diclofenac significantly suppressed the total oedema induced over 3 h with PGE_2_ (Figure 1(b)).

### 3.2. Effect of Extract on Clonidine-Induced Catalepsy

**Figure 2:**
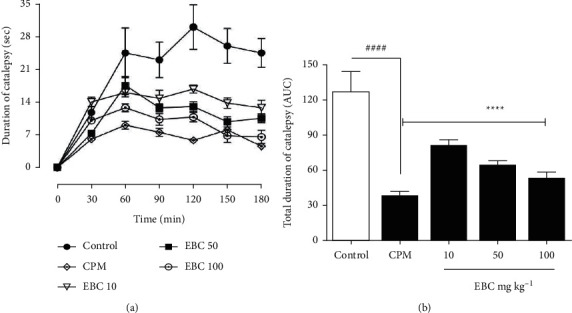
*Bombax costatum* extract inhibits clonidine-induced catalepsy. Data are presented as Mean ± S.E.M. (*n* = 5). ^####^*p* < 0.0001, chlorpheniramine-treated compared to vehicle-treated; ^*∗∗∗*^*p* < 0.001 and ^*∗∗∗∗*^*p* < 0.0001 extract-treated compared to vehicle-treated. (a) Mean duration of catalepsy. (b) Area under the curve (AUC) of the total duration of catalepsy.

Catalepsy was observed in all the treatment groups after clonidine (1 mg kg^−1^) was administered subcutaneously in the mice. Catalepsy was severest in the saline-control group as shown by displacement of the curve above all other treatment groups (Figure 2(a)). The administration of chlorpheniramine maleate (5 mg kg^−1^, ip) 30 min after induction of catalepsy significantly (*p* < 0.0001) reduced the mean duration of catalepsy (5.82 ± 1.12 s) when compared to the saline-control group (19.96 ± 3.95 s). Likewise, treatment with the extract (10, 50, 100 mg kg^−1^) significantly (*p* < 0.0001) suppressed the mean duration of catalepsy to 12.57 ± 2.16 s, 10.11 ± 2.07 s, and 8.14 ± 1.60 s, respectively, compared to 19.96 ± 3.95 s of the saline-treated control (Figure 2(a)). Once again, both chlorpheniramine maleate and the extract significantly (*p* < 0.0001) reduced the total duration of clonidine-induced catalepsy (Figure 2(b)).

### 3.3. Effect of the Extract on Haloperidol-Induced Catalepsy

**Figure 3:**
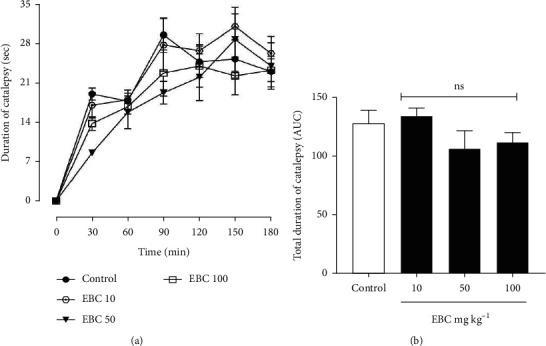
*Bombax costatum* extract inhibits haloperidol-induced catalepsy. Data are presented as mean ± S.E.M. (*n* = 5), ^*ns*^*p* > 0.05. (a) Mean duration of catalepsy. (b) Area under the curve (AUC) of the total duration of catalepsy.

Haloperidol (1 mg kg^−1^) was able to induce catalepsy in all groups, with the highest mean duration occurring in the saline-control group as shown by displacement of the curve above all other treatment groups (Figure 3(a)). Nonetheless, neither treatment produced a statistically significant reduction in catalepsy when compared to the saline-treated control. Treatment with the extract (10, 50, and 100 mg kg^−1^, p.o.), 1 h after haloperidol administration, exerted no significant (*p* > 0.05) inhibition on haloperidol-induced catalepsy, with a mean duration of catalepsy of 20.96 ± 4.00 s, 16.89 ± 3 s and 17.54 ± 3.26 s, respectively (Figure 3(a) and 3(b)).

#### 3.3.1. Effect of Extract on Rat Adjuvant-Induced Arthritis

**Figure 4:**
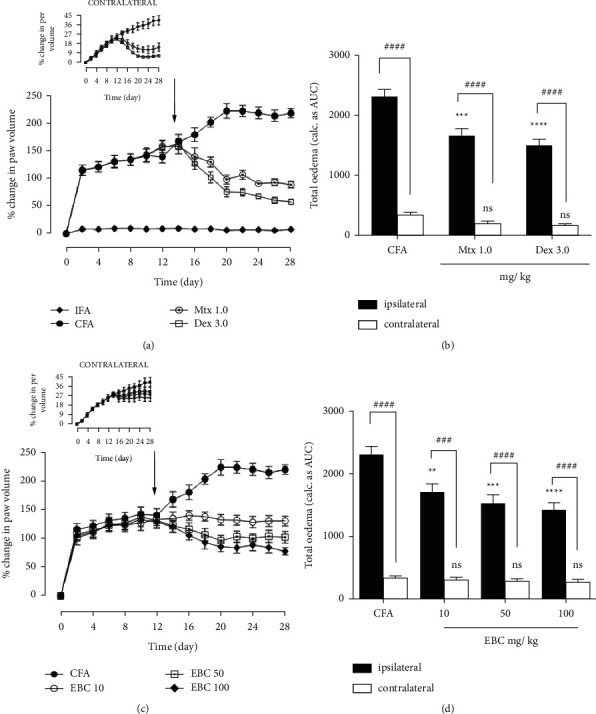
*Bombax costatum*extract inhibits adjuvant-induced arthritis. Values are mean ± SEM (*n* = 5). ^###^*p* < 0.001; ^####^*p* < 0.0001 compared to contralateral paw (two-way ANOVA followed by Tukey's post hoc test). ^*∗*^*P* < 0.05; ^*∗∗*^*p* < 0.01; ^*∗∗∗*^*p* < 0.001, and ^*∗∗∗∗*^*p* < 0.0001, respectively, compared to the control-treated group (one-way ANOVA followed by Turkey's post hoc test). Arrow indicates the day of drug commencement.

Complete Freund's adjuvant inoculation in rats resulted in inflammation of the injected paw (ipsilateral) which spread systemically to the noninjected paw (contralateral) with time. In this study (curative), daily administration of the extract (10, 50, and 100 mg kg^−1^, p.o.),which commenced on day 14 after injection of CFA,significantly suppressed the total oedema induced in rat paws (Figure 4(c))at the respective doses as compared to the arthritic control group. Similarly, the extract significantly decreased total inflammation by 26.14%, 34.07%, and 40.00% in a dose-dependent manner at 10, 50, and 100 mg/kg doses, respectively (Figure 4(d)). There was no significant change in paw volume of the IFA (nonarthritic group) (Figure 4(a)).

#### 3.3.2. Arthritic Indices and Radiological Assessment

**Figure 5:**
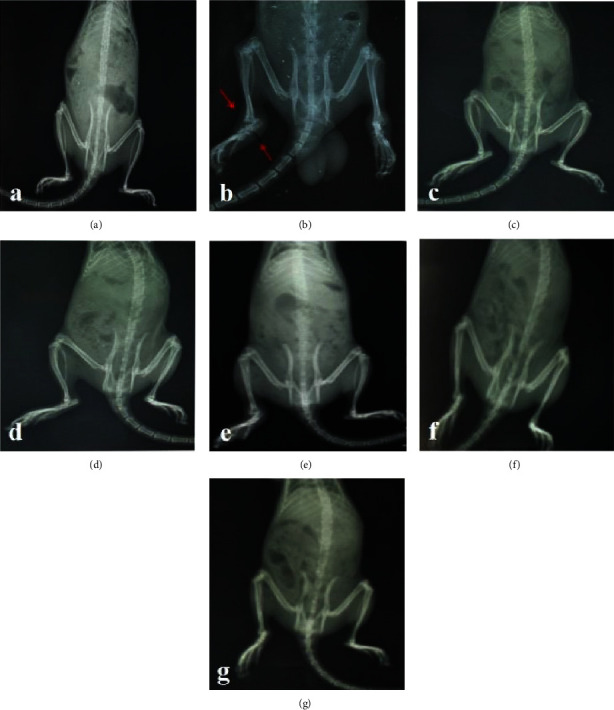
Effect of *Bombax costatum* extract on adjuvant-induced arthritis in rats. (a) Incomplete Freund's adjuvant (nonarthritic) group, (b) complete Freund's adjuvant (arthritic control) group,(c) methotrexate (1.0 mg/kg, ip), (d) dexamethasone (3.0 mg/kg, ip), and (e)–(g) extract (10–100 mg/kg, p.o.), respectively.

From the study, there were no characteristic signs of joint destruction, cartilage damage, and bone loss of both ipsilateral and contralateral limbs in the IFA (nonarthritic control) group (Figure 5(a)). Arthritic control rats (Figure 5(b)) showed the presence of severe joint destruction, bone loss, and cartilage damage in the ipsilateral limb spreading systemically to the contralateral limb over the 28-day period (Figure 5(b)). The extract at 100 mg kg^−1^ showed a significant inhibitory effect on bone loss, cartilage damage, and joint destruction in the ipsilateral limb compared to the CFA (Figure 5(b)). However, there was no significant inhibition observed in the contralateral limb as compared to the CFA (arthritic control group) (Figures 5(e)–5(g)). The respectively scored arthritic indices of the rats reflected the observations made from the radiographs as enshrined in the table (Table 1).

## 4. Discussion

Inflammatory response against invading pathogens or noxious stimuli is primarily aimed at eliminating tissue insults, resolving internal perturbations, and returning the body to homoeostasis [16]. Despite this protective intent, the inflammatory process, either acute or chronic, often goes awry and contributes to the pathogenesis of many diseases including rheumatoid arthritis, cancer, and cardiovascular diseases [17, 18]. The effect of the hydroethanol extract of the stem bark of *Bombax costatum* was therefore investigated in acute and chronic inflammatory models.

In this study, *Bombax costatum* extract inhibited prostaglandin *E*_2_ (PGE_2_)-induced paw oedema. The biosynthesis of prostaglandins is dysregulated and significantly increased in inflamed tissues and has been associated with the progression of the inflammatory response [1]. Specifically, dysregulated synthesis and degradation of PGE_2_, one of the most abundant prostaglandins, contributes to the development of processes that result in the development of all the cardinal signs of inflammation [18]. Interestingly, PGE_2_ has been shown to mediate the development of paw oedema following subplantar injection of collagen. This superfluous oedema occurs as a result of PGE_2_'s activity on the cognate EP_2_ and EP_4_ receptors [19, 20]. Similarly, EP_2_ and EP_3_ receptor stimulation by PGE_2_ produced during carrageenan-induced paw oedema and carrageenan-induced pleurisy, have been shown to cause inflammatory exudation [21]. From this study, it can be posited that *Bombax costatum* extract inhibits late-phase acute inflammatory response, through inhibition of PGE_2_ activity.

Additionally, the extract was also found to inhibit clonidine-induced catalepsy, a measure of the extrapyramidal effect of a drug. As such, the antioedematogenic effect of *Bombax costatum* extract may not only be mediated via inhibition of PGE_2_ activity, but the activity of histamine as well. To clarify, drugs that induce catalepsy achieve this effect through inhibition of dopaminergic transmission or the enhancement of histamine release in the brain [22]. One of such drugs is clonidine, a presynaptic *α*_2_-adrenoceptor agonist, that dose-dependently induces catalepsy via the enhancement of histamine release from mast cells in the brain [23]. This effect can be blocked by histamine H_1_ receptor antagonists but not H_2_ receptor antagonists [24]. Haloperidol, on the other hand, induces catalepsy through dopamine D_2_ receptor antagonism [25]. Thus, the lack of inhibition against haloperidol-induced catalepsy implies the absence of dopamine D_2_ activity by the extract. Thus, inhibition of catalepsy following clonidine administration suggests that *Bombax costatum* extract possesses histamine H_1_ antagonistic properties.

Moreover, mast cells are the major source of histamine in body tissues and are known to highly express histamine H_4_ receptors on their surfaces [26]. Stimulation of the H_4_ receptor on mast cells causes degranulation and mediates the proinflammatory response of histamine, through the activation of mitogen-activated protein kinases [27]. Additionally, activation of the receptor increases the expression of adhesion molecules, rearrangement of cytoskeleton, and changes in cell shape. Subsequently, these histamine-mediated responses increase immune cell migration to the site of inflammation leading to the development of the classic signs of inflammation such as oedema [28]. Furthermore, the H_4_ receptor-mediated activation of mast cells increases the stimulation of proinflammatory cytokine release, including the release of interleukin-6 and tumour necrosis factor-*α* [29, 30]. For this reason, the antihistaminic effect of *Bombax costatum* extract may possibly indicate the ability to inhibit mast cell degradation. However, further investigation is needed to confirm this proposed mast cell stabilizing property of the extract during acute inflammation.

Likewise, *Bombax costatum* extract was identified to suppress chronic inflammation associated with adjuvant-induced arthritis. This model is a chronic inflammatory model widely utilized in the preclinical screening of anti-inflammatory agents used in the management of rheumatoid arthritis [31, 32]. Injection of heat-killed *Mycobacterium tuberculosis* induces autoimmune inflammation that is characteristically like an immunological response in human rheumatoid arthritis. In the same way, adjuvant-induced arthritis is characterized by cellular infiltration of the synovial membrane and joint destruction which resembles the human disease [33].

In this study, treatment with *Bombax costatum* extract reduced the synovitis, swelling, and joint inflammation associated with adjuvant-induced arthritis. Furthermore, the extract reduced joint inflammation with minimal joint deformation and narrowing. This antiarthritic effect of the extract can be attributed in part to the inhibition of PGE_2_ synthesis and activity, as previously shown. This is because, PGE_2_ produced in rheumatoid synovium, via activation of EP_4_ receptors, contributes to IL-6 production and joint destruction [34, 35]. This is supported by findings that mice that have EP_1_, EP_2_, or EP_3_ receptors but are deficient in EP_4_ show an attenuated response to joint inflammation, with significantly lower levels of IL-6 and IL-1 and a dramatic reduction in the disease severity [36]. Thus, through the inhibition of PGE_2_-EP_4_ activity, the extract inhibited the development and progression of arthritic features following injection of complete Freund's adjuvant (CFA).

Like human rheumatoid arthritis, rat adjuvant-induced arthritis is generally a systemic illness, with inflammation that spreads and affects tarsal and phalangeal joints, even of the uninjected paw, after 11–16 days [37]. This is known as polyarthritis. Interestingly, treatment with *Bombax costatum* extract was able to inhibit the development of polyarthritis on day 13 post-CFA injection. This implies that the extract can suppress and limit the spread of joint inflammation to unaffected joints. This anti-inflammatory activity of *Bombax costatum* extract may be associated with the presence of catechin-7-O-glucoside [38]. The pharmacological properties of catechins have been extensively studied to reveal their anti-inflammatory effects. For instance, catechins have been shown to inhibit TNF-*α* and NF-*κ*B pathways and the release of nitric oxide in acute and chronic inflammation [39, 40]. Thus, in human rheumatoid arthritis, *Bombax costatum* extract may serve as a source of drug leads that can inhibit or delay the onset of polyarthritis and other associated systemic manifestations, such as ocular, nasal, dermal, and metabolic manifestations [37].

## 5. Conclusion

Given the above, the hydroethanolic extract of *Bombax costatum* is effective against acute and chronic inflammation. This study has shown for the first time that, *Bombax costatum* extract suppresses oedema associated with acute inflammation through the inhibition of the action of prostaglandin *E*_2_ and histamine. Moreover, our findings also suggest that *Bombax costatum* extract may be a potential source of lead compounds, for use in the management of rheumatoid arthritis.

## Figures and Tables

**Table 1:** Arthritic indices for rats in adjuvant-induced arthritis evaluated from radiographs.

Groups	Radiological index	
	Ipsilateral	Contralateral
IFA	0	0

CFA	2.60 ± 0.25	2.40 ± 0.25

Methotrexate		
1.0 mg/kg	0.5^*∗∗∗*^	0.9^*∗∗∗*^

Dexamethasone		
3.0 mg/kg	0.59^*∗∗∗*^	0.99^*∗∗∗*^

Extract		
10 mg/kg	2.40 ± 0.24	2.20 ± 0.20
50 mg/kg	1.80 ± 0.20	2.00 ± 0.31
100 mg/kg	1.40 ± 0.24^*∗*^	1.80 ± 0.20

One-way ANOVA followed by Turkey's post hoc test. All data are presented as mean ± SEM (*n* = 5). ^*∗*^*P* < 0.05, ^*∗∗*^*p* < 0.01, and ^*∗∗∗*^*p* < 0.001 compared to the complete Freund's adjuvant control group. CFA, complete Freund's adjuvant; IFA, incomplete Freund's adjuvant.

## Data Availability

All data are available on request.
